# Identifying the challenges to successfully teaching about genetic diversity among Japanese junior high school students

**DOI:** 10.1177/2050312120960656

**Published:** 2020-09-20

**Authors:** Natsu Kohama, Hiromi Kawasaki, Chieko Kukinaka, Hiromi Goda, Md Moshiur Rahman

**Affiliations:** 1 Program of Health Sciences, Graduate School of Biomedical and Health Sciences, Hiroshima University, Hiroshima, Japan; 2 School and Public Health Nursing, Graduate School of Biomedical and Health Sciences, Hiroshima University, Hiroshima, Japan; 3 Faculty of Life Sciences, Kumamoto University, Kumamoto, Japan; 4 International Health & Medical Care, Graduate School of Biomedical and Health Sciences, Hiroshima University, Hiroshima, Japan

**Keywords:** Genetic education, inclusive education, diversity, school health, school health teacher

## Abstract

**Objectives:**

Creating a diverse and inclusive symbiotic society is specified in the sustainable development goals. In a symbiotic society, support for those who need, it is called “reasonable support.” However, it is unclear in the classroom that many children understand “reasonable” as a consideration to support children with special needs. The aim of this study is to identify the actual understanding of junior high school students and the challenges related to genetic diversity through school health teachers in readiness for developing a symbiotic society.

**Methods:**

A focus group interview was conducted for five school health teachers working in public junior high schools to identify the perspectives of their feeling about the current understanding of children in regard to genetics and diversity. Participants were recruited who agreed to engage voluntarily in this research. A qualitative descriptive design was used in this study.

**Results:**

The results of the analysis revealed three categories consisting of 67 codes and 10 subcategories. Three categories were identified: I—understanding the heterogeneity and diversity of children’s ambivalent minds; II—limitation of school health teachers’ involvement in genetics and diversity-related issues; and III—importance for children to understand heterogeneity and diversity to build life skills.

**Conclusions:**

School health teachers remarked on the limitations of learning and teaching genetics and diversity. They paid attention to the flexibility of a child. It suggests that the purpose of genetic education is to develop children’s life skills with the flexibility to live in the future. There is a need to consider new genetic education for school health teachers and students to learn about diversity.

## Introduction

In 1994, the United Nations Educational, Scientific and Cultural Organization (UNESCO) and the Government of Spain held the “World Conference on Special Need Education: Access and Quality” and adopted “The Salamanca Statement and Framework for Action on Special Needs Education,” which stated that schools were the most effective resources to achieve education and build an inclusive society for all.^1^

According to the “Universal Declaration on the Human Genome and Human Rights” announcement, it was declared that “Everyone has a right to respect for their dignity and for their rights regardless of their genetic characteristics. That dignity makes it imperative not to reduce individuals to their genetic characteristics and to respect their uniqueness and diversity.”^2^ However, the reality is that peers of students with disabilities sometimes express extremely negative attitudes toward such students in classrooms^3^ and many of them with disabilities have experienced bullying.^4,5^ Some parents of non-disabled children are concerned about their children being negatively affected by having children with disabilities.^6^ Siblings of children with genetic diseases are not able to receive an adequate education due to lack of understanding from their surroundings.^7^ There is discrimination against genetic diseases. Some Japanese people believe that taking care of a genetic disease is personal management, and society does not need to take special care.

In Japan, the government said it is important to promote inclusive education. This is to create a diverse and inclusive symbiotic society as indicated in the sustainable development goals (SDGs).^8^ In a symbiotic society, children with disabilities are supported to study together. Specific support is called reasonable accommodation. However, it is not clear in the classroom that children understand “reasonable” as a consideration of supporting children with special needs. Each of the children needs to understand children with special needs as inclusive diversity. Japanese junior high school students learn about genetics in science. The concept of genetics is useful for understanding each person’s differences. When children learn the right knowledge, there is a great impact globally for understanding diversity.^9^ Education is important for people to fully understand diversity and genetics.

In order to provide inclusive education, we first thought that we needed to clarify the links between students’ genetics and real life and the idea of diversity. However, it is the school health teacher who helps students to understand genetic diversity in a comprehensive manner. In other words, it is important to identify how school health teachers are working in public junior high schools to perceive and listen to children’s reactions toward genetics and diversity and identify issues related to school health teachers dealing with students. The purpose of this study is to identify the actual situation of children and surrounding environment related to the genetic diversity through school health teachers.

## Methods

### Design

A qualitative descriptive design was used to investigate the opinions on genetic disabilities and diversity at the schools where school health teachers were employed and to identify the perspectives of school health teachers regarding how they feel about the current situation of children in regard to genetics and diversity. Similar statements with same meaning were extracted from the data. The concept was optimized after carefully examining the collected data. The relationship between the categories and the integrity of the overall results were examined, and when it was judged as there was no shortage, theoretical saturation was determined.

### Study participants

The participants in this study were five school health teachers working in public junior high schools who gave consent to participate in this study. They were enrolled using a snowball method, which was introduced one after another by one teacher. Seven teachers agreed to participate; two teachers failed to join in the interview because the interview schedule did not match with them. The authors and the first school health teacher knew each other but met other teachers for the first time. They work in the local municipalities in a prefectural urban setting in Japan. The school health teachers are full-time teachers and existing in the Japanese educational system. Their job responsibilities are to provide primary health care and health promotion activities utilizing the knowledge of nursing for children who are attending school.^10^ Though nursing license is not a mandatory requirement, a few of them have it anyway. They maintain a close contact children’s class teachers and guardians to ensure good health of children. School health teachers are not responsible for teaching other academic subjects and evaluating grades. Hence, children often talk freely to school health teachers. That is why, we have interviewed school health teachers. As genetics is taught in junior high school in science classes, respective teachers were targeted considering the possibility of healthy discussions about the human genetic diversity in classrooms. All the school health teachers were women, and the mean of work experience was 17.2 years.

### Data collection

Participants were recruited who agreed to involve in this study. A focus group interview (FGI) was conducted in August 2017. An author was supported by a masters-level student to keep records. The facilitator was a female school health teacher who holds a license for a public health nurse with PhD. She had experiences in conducting semi-structured interviews and FGI. The FGI lasted 52 min on the same day in a University lecture room during summer vacation. A moderator led the FGI of all participants based on an interview guide (Supplemental Appendix 1). The location of the FGI was chosen to ensure privacy. With the permission of participants, a voice recorder was used for recording, and a transcript was created. During the interview, the anonymity of the participants was assured using numbers instead of real names of participants on the cards. The interview guide focused on the following: (1) experiences in supporting children after being asked genetics-related questions or providing consultation; (2) difficulties or challenges with responding or providing assistance to genetics-related matters; (3) the connection between genetics education during science lessons and the real lives of children; (4) education of human diversity in a world where people have a choice to learn and access their genetic information; and (5) future expectations of school health teachers in regard to responding to genetics-related questions and consultations.

### Data analysis

After conducting the FGI, a qualitative descriptive analysis was performed based on the created transcript. Based on the same semantic content, we classified them from code to the subcategory, and then to category and abstraction. Eventually, a category theme was generated. Participants were not checked at the data analysis stage, but confirmed the final data. The transcript was not returned.

### Trustworthiness

To confirm the content of the results of analysis and improve the validity and reliability of data, three investigators repeatedly engaged in discussions about the analysis results. An expert researcher in qualitative studies supervised this study.

### Outline of Japanese junior high school education system

Japanese junior high school education system is determined by the Ministry of Education, Culture, Sports, Science and Technology (MEXT). For public schools, there is a standard national curriculum. The number of hours and contents of teaching are guided and fixed by the MEXT. The annual education program should be developed to cover 35 or more school weeks for teaching all subjects. However, the daily hours for the school lunch, recesses, and other activities need to be properly determined at each school level. The school is also instructed by the MEXT to create interconnections between different subjects.

Health education is included in health and physical education lessons. The health sector provides health promotion, nutrition, and disease prevention information. In the field of physical education, students will learn swimming, ball games, and traditional martial arts.

Science education is divided into the area of learning the characteristics of materials, biology, and environment. The latter includes respect for life, conservation of the natural environment, and radiation. These programs are linked between the national goals and the SDGs.

### Ethical approval

The approval was obtained from the Hiroshima University Ethics Committee (No. E800-1). In this study, written informed consent was obtained from all participants after explanations of the study methods, purpose, and meaning that no negative consequences would be incurred by refusing or withdrawing from the study, privacy would be protected, and the participant could withdraw at any time without penalty.

## Results

The results of the analysis revealed three categories consisting of 67 codes and 10 subcategories (Table 1).

**Table 1.** How school health teachers perceive children’s reactions toward genetics and diversity and think about genetics-related concerns.

Category		Subcategory
I	Understanding the heterogeneity and diversity of children’s ambivalent minds	1. Children’s aversions and discriminatory feelings toward heterogeneity
		2. Children can understand and accept diversity
II	Limitation of school health teachers’ involvement in genetics and diversity-related issues	1. There is some hesitation in intruding on children’s personal information
		2. It is important to respect the opinions and wishes of the parents
		3. We communicate to children information that is familiar and general in content
		4. Not confident to explain as it may greatly impact a child
		5. There is a sense of difficulty in explaining genetic concepts to children
		6. Conflicts over genetic education that arise by taking a child’s perspective
III	Importance for children to understand heterogeneity and diversity to build life skills	1. The purpose of teaching genetic education is to nurture the children’s life skills
		2. It is important to understand diversity through familiar situations

### Category I: understanding the heterogeneity and diversity of children’s ambivalent minds

The school health teachers sensed the ambivalence of the children at school toward understanding genetics and diversity. The children’s responses to understanding diversities are described in two ways:

#### Children’s aversions and discriminatory feelings toward heterogeneity

School health teachers observed that children in school sometimes displayed negative attitudes toward children with disabilities and illnesses, harboring discriminatory feelings toward foreign elements. The children themselves disliked being different from others:

I heard children calling other names such as “retard.” (Participant #1)

I sensed that children did not want others to think that they were different. (Participant #3)

. . . When the children were told that “the illness was caused by innate genetic factors,” they wondered if the illness was contagious. There was a feeling of discrimination, even among adults. (Participant #4)

#### Children can understand and accept diversity

There was a school health teacher who recalled the behavior of children leading lives oblivious to their disabilities:

. . . Children with illnesses and disabilities are leading normal lives, so I have never encountered any problems. There is a high probability of finding children with illnesses and disabilities in schools. The children had been together from a young age, so they probably learned about the illnesses and genetic issues during their elementary school years. (Participant #5)

### Category II: limitation of school health teachers’ involvement in genetics- and diversity-related issues

Six subcategories emerged in relation to school health teachers becoming involved in genetics- and diversity-related issues.

#### There is some hesitation in intruding on children’s personal information

School health teachers said that they were hesitant to explain genetic issues when they felt it may relate to personal issues:

I am conscious not to expose personal information. I think blood type is also personal information. I did not explain to students that a child’s blood type is determined by the parents’ blood types. I only provided answers to questions asked by the students. (Participant #2)

Children may believe that they are related to the parents by blood; however, they may actually not be blood-related. (Participant #2)

It is difficult to give specific answers to questions related to genetics as some children know that their parents have remarried. (Participant #2)

School health teachers were respectful of the children’s circumstances surrounding their birth. They stated that explanations were confined within limited boundaries so as not to affect the lives of the children.

#### It is important to respect the opinions and wishes of the parents

School health teachers brought up the relationship with parents in relation to personal information:

I once asked a child with color blindness if he or she had ever gone to a hospital to get it checked. The child had not been to the hospital. The parents also seemed like they did not want to take the child to the hospital. (Participant #3)

First of all, the issue is whether we can talk to the child about the illness or genetic issue. Parental consent is needed to be able to talk to other students about a child’s illness. (Participant #4)

#### We communicate to children information that is familiar and general in content

When school health teachers are asked to explain or conduct consultations on genetics- and diversity-related questions, they can describe as long as the explanations are general or brief overviews:

I may be able to give a general explanation about Down’s syndrome to children, for instance. (Participant #1)

When I was asked about blood types, which the children had learned about in science class, I explained using some easy and familiar topics with diagrams. (Participant #2)

#### Not confident to explain as it may greatly impact a child

School health teachers are aware of their role to provide consultation and explanations related to genetic diseases and disabilities when asked. School health teachers revealed that they were not confident explaining topics on genetics and diseases to children:

Questions are asked out of the blue. The questions come out of nowhere without us being prepared. However, if school health teachers know that these things could happen, they could say “let me think about it” and handle the situation without providing an immediate response. (Participant #2)

I could not give an appropriate answer when a child asked, “His or her condition is caused by genes, right? Is it the parents’ fault?” (referring to a child with Down’s syndrome). (Participant #4)

When explaining a disease to a child, the way I explain it will greatly impact the child. Providing an explanation on the spot is a very difficult thing to do and I cannot do it. (Participant #4)

#### There is a sense of difficulty in explaining genetic concepts to children

School health teachers asserted that it is difficult to explain genetic concepts as part of teaching diversity. Genetic education for children focusing on both scientific and moral issues is more difficult:

Because topics on genetics taught in science are not linked to moral lessons, genetics education is not effective unless the timing for conducting the science and moral lessons are coordinated to occur close together. (Participant #1)

Methods of teaching genetic education are difficult. (Participant #5)

Combining knowledge acquired through science with moral concepts is a very challenging undertaking for children. (Participant #5)

#### Conflicts over genetic education that arises by taking a child’s perspective

School health teachers raised the issue that there are conflicts, which occur from teaching genetics to children. School health teachers are concerned about the psychological implications for children. They mentioned that learning about genetics may raise fear and anxiety toward the future in children:

I feel that children do not need to learn about difficult genetic topics when they are young. (Participant #1)

I want to talk to the children about genetics and inform them that the possibilities of developing congenital disorders and diseases can be determined through genetics. I want the children to be mentally prepared. However, if the children are going to live their lives worrying that “one day they might become ill,” it is probably better not to talk to them about genetics. (Participant #2)

The children may become disillusioned if they learn that they could become ill in the future. In fact, I am also scared. (Participant #5)

### Category III: importance for children to understand heterogeneity and diversity to build life skills

After discussing the children’s ambivalent responses toward diversity and the limitations faced by school health teachers, they voiced the need for future education that focuses on understanding heterogeneity and diversity to nurture the children’s life skills. Two subcategories related to this opinion emerged.

#### The purpose of teaching genetic education is to nurture the children’s life skills

School health teachers suggested that understanding diversity in genetics education was for children to nurture their life skills:

Children need to acquire the ability to make their own choices and decisions. Or perhaps, when in trouble, instead of worrying alone, without hesitation, they need to ask someone for help to resolve their problems. (Participant #2)

When one is confronted with various problems, instead of relying on simple tests and purchasing products, it is important to first think if these are really needed. They then need to think what they will do once the test results arrive. I think that acquiring the ability to think and make judgments at a young age will allow them to lead a safer life. (Participant #1)

#### It is important to understand diversity through familiar situations

School health teachers believe that for children to understand diversity, it is important to do so through familiar situations. Similarly, school health teachers need to have adequate knowledge and understanding of genetics:

. . . Genetics-related issues are problems that living humans face in real life. We are not talking about frogs, these are human issues . . . (Participant #1)

Learning from familiar topics will also allow them to learn about the emotional aspects . . . (Participant #1)

School health teachers will need genetic knowledge in the future. (Participant #3)

## Discussion

The school health teachers observed the ambivalent responses from the children toward understanding diversity. They perceived the children’s inconsistent reactions as children have the ability to understand diversity; however, genetic education is needed to promote understanding of diversity. In other words, the school health teachers concluded that the children were fully capable of understanding diversity.

While the school health teachers felt that the children were completely able to understand diversity, they also felt that there was a limit for school health teachers to involve in genetics- and diversity-related issues. One of the reasons for this context is because genetics- and diversity-related issues contain personal information.^11^ In another study, teachers described that some guardians did not feel comfortable to communicate to the school about their children’s sickness and the actual health conditions.^12^ School health teachers in this study mention that it is necessary to respect not only the children but also the parents’ thought including their doubtfulness and expectations. School health teachers know that they must pay careful attention when teaching and providing supports for the understanding of diversity, as insufficient explanations and biased information may hurt those concerned.^13^ In a genetic problem, they already know that the adjustment in the family and the protection of rights are also important.

The second concern that has been identified as the required genetic education is the influence that school health teachers can provide to the children. However, they are not confident enough in explaining specific cases with technical contents, such as in the case of genetic diseases. It is expected to ensure effective knowledge transfer by avoiding the use of over technical and medical jargon.^14^ School health teachers worry about their explanation or choices of words influencing child’s understanding and opinions. They are concerned to learn and resolve the methods and communication strategies of the provision of genetic information sharing. The school health teacher’s curriculum needs to include these contents.

The third concern is the difficulty in teaching genetics for the purpose of understanding diversity. Because of its theoretical nature, genetics is difficult to conceptualize; hence, children must have sufficient concepts of all subjects related to genetics to understand clearly.^15^ Only school education is stipulated by law, and the standard for compulsory education is provided. Areas related to this article are science, health and physical education, and morals. School health teacher is mainly responsible for health education together with physical education teacher. The MEXT, Japan, stipulates related topics to be learned in junior high school regarding genetics (Table 2). These are “cell division and biological growth,” “genetic regularity and genes,” and “diversity and evolution of species.”^16^ Nevertheless, school health teachers believe that children also need to learn the morality of mind to understand diversity. As a consequence, children learn the basic contents of morality during their first 6 years of elementary study. For example, they learn the concept of politeness, humility, acceptance, cooperation, the dignity of life, and keeping the rules of society.^17^ In junior high school, this learning will develop basics and proceed in consideration of the characteristics of adolescence. Based on our relationship with society, education will be developed to focus on our way of life as a human being and our relationship with society. Therefore, children learn to have empathy and fairness toward others through the moral education.^18^ Decision-making on issues related to genetics and diversity is greatly influenced by moral aspects.^19^ Teachers thought that diversity could be better understood by combining knowledge and moral, because children need to acquire enough knowledge and have deep moral education. These are to foster morality which is the foundation for students to think about life as a human being, act based on independent judgments, and live a better life with other people as an individual. Better living is related to the global goals of the good health (SDG-3), gender equality (SDG-5), and reduced inequalities (SDG-10), and adopted in the Japan national policy.^8^ To reduce inequalities and understand diversity, the government is promoting SDGs goals typically in the junior high school education.

**Table 2.** Course of study in Japanese junior high school on science content related to genetics: Continuity of life.

Unit	Goals and content	Notes
Cell division and biological growth	Students observe how creatures grow. Students understand the characteristics of sexual and asexual reproduction Students understand that parental traits are transmitted to their offspring as the organism grows	1. Teachers mention that chromosomes are replicated 2. Sexual reproduction mechanisms should be linked to meiosis
Genetic regularity and genes	Based on the results of the mating experiment, students will understand the regularity of the parent’s trait when it is transmitted to the offspring Students understand that the trait is transmitted from parent to offspring via genes on the chromosomes and the rules of segregation	1. Focusing on one trait, the teacher instructs the student on how the trait is transmitted to the offspring and grandchildren 2. Students find the rules of heredity and understand how they work
Diversity and evolution of species	By comparing existing creatures and fossils, students understand that a wide variety of existing creatures is the result of the transformation of past creatures over a long period of time, in connection with body construction	1. Teachers should deal with evidence of evolution and specific examples of evolution 2. Teachers should note that living creatures have characteristics that favor living in their habitat 3. Teachers should also note that traits may change due to changes in genes

Source: The standard for education curriculum announced by the Ministry of Education, Culture, Sports, Science and Technology.

The difficulty of genetic education lies not only in teaching but also in learning. Genetic knowledge and information for children may affect many aspects of their future lives.^20^ Although the previous studies reported that there was limited genetic information on the adverse psychological association with individual children, some of them found more concerned about their health and experienced familial problems like distress, blame, and prejudice.^21^ School health teachers considered that children may become anxious for their future by learning about genetics. Therefore, it is not easy for school health teachers to teach genetics and diversity. Teachers should find out precisely how children will perceive genetic information and keeping in mind that children may not necessarily cope well with information as they could develop genetic diseases in the future.^22^ School health teachers need to take children’s feelings into consideration. During the FGI, the school health teachers felt troubled and conflicted about children learning on genetics at a young age when viewing the situation from both the educators’ and children’s perspectives. In regard to the practice and knowledge of the genetic, it is important to know the limits of the school health teachers themselves. It has suggested that self-improvement is needed.

School health teachers believe that genetic education for understanding diversity should teach children about life skills. According to the World Health Organization (WHO) definition “Life skills are abilities for adaptive and positive behaviour that enable individuals to deal effectively with the demands and challenges of everyday life.”^23^ In fact, life skills support people through the competencies and skills for the healthy and productive life, coping with realities, thinking deeply, solving problems, decision-making, communicating efficiently, and developing a healthy relationship.^24^ In this way, life skills include abilities to sort out essential information and obtain objective information to be capable of making decisions on their own.

With the advancements in genetic technology, it is important for the current generation of children to understand about the science, benefits, risks, and ethical issues and to be prepared to make personal decisions about their own genomes and health.^25^ In addition, life skills should be learned from familiar experiences. School health teachers believe that knowledge related to genetics and diversity should not only be acquired but also needs to put into practice. While leveraging the school health teachers’ social resources, they also need to learn to acquire state-of-the-art knowledge. In inclusive education, curriculum planners need to consider new genetic education for school health teachers and students to learn the diversity.

## Limitations

The main limitation of this study was that it only targeted school health teachers in an urban setting in Japan. The participants of this study are school health teachers working in a specific area of a public junior high school. They were collected in a snowball manner. It may be influenced by the characteristics of the status and experience. A study focusing on not only school health teachers but also general teachers should be conducted in the future.

## Conclusion

School health teachers notice children’s ambivalent responses toward genetics- and diversity-related issues. Moreover, there is a limitation on the involvement of school health teachers in these issues. It is difficult for children to completely understand genetics and diversity in traditional education. School health teachers believe that new education for understanding heterogeneity and diversity should be aimed at nurturing children’s life skills. They emphasize that it is good to have the life skills to deal with various complicated problems. And they suggest that the materials of genetic education are to include familiar contents. It will make important and relevant connections to real life.

## Supplemental Material

Additional_file_1_Interview_guide – Supplemental material for Identifying the challenges to successfully teaching about genetic diversity among Japanese junior high school students Click here for additional data file. Supplemental material, Additional_file_1_Interview_guide for Identifying the challenges to successfully teaching about genetic diversity among Japanese junior high school students by Natsu Kohama, Hiromi Kawasaki, Chieko Kukinaka, Hiromi Goda and Md Moshiur Rahman in SAGE Open Medicine

Additional_file_2_COREQ_Checklist – Supplemental material for Identifying the challenges to successfully teaching about genetic diversity among Japanese junior high school students Click here for additional data file. Supplemental material, Additional_file_2_COREQ_Checklist for Identifying the challenges to successfully teaching about genetic diversity among Japanese junior high school students by Natsu Kohama, Hiromi Kawasaki, Chieko Kukinaka, Hiromi Goda and Md Moshiur Rahman in SAGE Open Medicine
