# Supplementary material for: Identifying the challenges to successfully teaching about genetic diversity among Japanese junior high school students
Source: SAGE Open Med. 2020 Sep 20;8:2050312120960656. doi: 10.1177/2050312120960656 (PMC7509707; doi:10.1177/2050312120960656)
Supplement: Additional_file_1_Interview_guide – Supplemental material for Identifying the challenges to successfully teaching about genetic diversity among Japanese junior high school students [file Additional_file_1_Interview_guide.docx]

Interview guide for school health teacher

Did you think it was difficult to answer the consultation?

Do you think students relate the sciences of genetics to real life?

Companies are now offering services to examine genes. The era of choosing to know for yourself is coming. How do you answer if a student asks you to take a genetic test?

Did you think it was difficult to answer the consultation?

What skills are needed to solve the questions of genetic consultants?

When should the skills be developed as a public health nurse?

What did you answer at that time?

**You thought to be involved in genetic**: Did you think it was difficult to answer the consultation?

**You do not think as a genetic:** What are the reasons that you thought that there is no relation to the genetic?

Can you solve it?

Yes

No

Have you had any consultation or support related to genetic?

What kind of consultation?

What do you imagine about genetic counseling?

Is there any possibility that students will consult you, such as being genetic?

Yes

No
